# Toward quantitative CEST imaging of glutamate in the mouse brain using a multi‐pool exchange model calibrated by ^1^H‐MRS

**DOI:** 10.1002/mrm.30353

**Published:** 2024-10-24

**Authors:** Cécile Maguin, Eloïse Mougel, Julien Valette, Julien Flament

**Affiliations:** ^1^ Molecular Imaging Research Center, Laboratoire des Maladies Neurodégénératives Université Paris‐Saclay, Commissariat à l'Energie Atomique et aux Energies Alternatives, Centre National de la Recherche Scientifique Fontenay‐aux‐Roses France

**Keywords:** ^1^H‐MRS, Bloch‐McConnell fitting, CEST, glutamate, quantification, quantitative CEST

## Abstract

**Purpose:**

To develop a CEST quantification model to map glutamate concentration in the mouse brain at 11.7 T, overcoming the limitations of conventional glutamate‐weighted CEST (gluCEST) contrast (magnetization transfer ratio with asymmetric analysis).

**Methods:**

^1^H‐MRS was used as a gold standard for glutamate quantification to calibrate a CEST‐based quantitative pipeline. Joint localized measurements of Z‐spectra at B_1_ = 5 μT and quantitative ^1^H‐MRS were carried out in two voxels of interest in the mouse brain. A six‐pool Bloch‐McConnell model was found appropriate to fit experimental data. Glutamate exchange rate was estimated in both regions with this dedicated multi‐pool fitting model and using glutamate concentration determined by ^1^H‐MRS.

**Results:**

Glutamate exchange rate was estimated to be ˜1300 Hz in the mouse brain. Using this calibrated value, maps of glutamate concentration in the mouse brain were obtained by pixel‐by‐pixel fitting of Z‐spectra at B_1_ = 5 μT. A complementary study of simulations, however, showed that the quantitative model has high sensitivity to noise, and therefore, requires high‐SNR acquisitions. Interestingly, fitted [Glu] seemed to be overestimated compared to ^1^H‐MRS measurements, although it was estimated with simulations that the model has no intrinsic fitting bias with our experimental level of noise. The hypothesis of an unknown proton‐exchanging pool contributing to gluCEST signal is discussed.

**Conclusion:**

High‐resolution mapping of glutamate in the brain was made possible using the proposed calibrated quantification model of gluCEST data. Further studying of the in vivo molecular contributions to gluCEST signal could improve modeling.

## 1 INTRODUCTION

CEST is an MRI‐based molecular imaging technique offering great potential. Unlike ^1^H‐MRS that is limited by its intrinsic low sensitivity, CEST imaging can offer a valuable alternative to map spatial distribution of several brain metabolites with a good resolution. This technique indirectly detects dilute molecules by exploiting their exchange of labile protons with bulk water. Exchangeable protons, such as amine (–NH_3_^+^) or amide (–NH), have a resonance frequency different from that of free water and can be selectively saturated using an RF pulse. Because the decrease of water signal because of magnetization exchange is directly related to the concentration of exchanging protons, it is possible to map the spatial distribution of several molecules of interest, especially brain metabolites involved in several aspects of normal brain functions including cognition, memory, and learning. For instance, CEST can be applied to map glutamate (Glu), a neurotransmitter naturally abundant in the brain, which exhibits a CEST resonance at 3 ppm.^1^ Various studies have already demonstrated the potential of Glu‐weighted CEST, gluCEST, to map Glu in both rodent and human brains. Thanks to the excellent spatial resolution offered by CEST, previously reported gluCEST images allow for the identification of main brain regions and even finer structures, like hippocampus pyramidal assembly.^2^ Furthermore, gluCEST has proven to be of particular interest to monitor neurometabolism and detect neurological pathologies. In rodents, gluCEST has been used to study neurodegenerative diseases like Huntington's,^3^, ^4^ Alzheimer's,^5^ or Parkinson's,^6^, ^7^ but also afflictions like cerebral ischemia,^1^ epilepsy,^8^ or traumatic brain injuries.^9^ In the human brain, gluCEST studies have shown that Glu might be a unique biomarker to detect tumor,^10^ epilepsy,^11^, ^12^ or encephalitis,^13^ sometimes proving to be more reliable than traditional MRI. GluCEST has also been used to study disorders on the psychosis spectrum^14^, ^15^ and multiple sclerosis.^16^

However, the interpretation of the conventional gluCEST contrast, which is based on the measurement of the magnetization transfer ratio with asymmetric analysis (MTR_asym_) metric, can be confounded by concomitant effects such as the modification of the magnetization transfer^17^ (MT) or potentially non‐negligible downfield nuclear Overhauser effects (NOE), even at high B_1_ saturation power. Furthermore, even when using less biased CEST metrics, such as inverse Z‐spectrum (MTR_Rex_),^18^ with proper removal of MT and NOE effects, gluCEST contrast is suspected to be contaminated by the contributions of other metabolites like amides or amines,^19^ or by some amino‐acids like aspartate.^20^ Even when choosing the optimal saturation parameters for Glu‐targeted imaging, over 40% of gluCEST contrast might be related to other CEST agents.^19^ To top it off, Glu exchange dynamics are known to be extremely sensitive to chemical environment (Figure S1 and Tables S2, and S3), temperature or pH. As changes of pH have already been reported in various stress conditions,^21^ it is important to account for this effect for reliable evaluation of Glu concentration in vivo.

Consequently, to increase the reliability and specificity of gluCEST imaging, quantification methods to determine the correct Glu concentration from CEST measurements are of utmost interest. Quantitative CEST (qCEST) has been an emerging field in the recent years,^22^ both for the in vivo quantification of exchange rates and of metabolite concentrations relative to water. Approaches such as Lorentzian decomposition of the Z‐spectrum (or polynomial and Lorentzian lineshape fitting) have been proposed,^23^, ^24^ with successful application to slow‐exchanging proton pools.^25^ However, the Z‐spectrum lineshape at 5 μT, which is a commonly used saturation power in gluCEST studies,^4^, ^26^ is broad and the specific peak related to Glu hardly distinguishable from other contributions, which makes it hard to fit properly with PLOF. Other more time‐efficient quantification methods, like quantification through varying saturation power or time (QUESP or QUEST)^27^ are theoretically applicable to rapidly exchanging protons like amine protons of Glu,^28^ but they require specific and uncontaminated CEST contrast, which is not the case of gluCEST, especially in vivo where the signal is mixed together with contributions of other CEST agents.^19^, ^20^ Hence, a quantitative gluCEST pipeline was developed based on the gold‐standard approach, which is the fitting of Z‐spectra by Bloch‐McConnell simulations using a CEST model including several proton‐exchanging pools resonating at different frequencies.

The difficulty with this approach is the large number of unknown variables, including proton fractions and exchange rates of each pool. In particular, Glu exchange rate (k_ex_^Glu^), which is a key parameter for gluCEST quantification, has never been accurately measured in vivo. Although it has been extensively studied in vitro,^1^, ^19^, ^29^, ^30^ exchange dynamics of Glu may be different in vivo, just like it was recently reported for Cr.^31^ The lack of knowledge on in vivo k_ex_^Glu^ significantly limits the possibility of doing proper quantitative gluCEST imaging. One aim of this work is, therefore, to obtain an estimation of Glu's exchange rate in the mouse brain, and then use it as a tuned value to efficiently perform quantitative gluCEST imaging.

To develop this calibrated CEST model, quantitative ^1^H‐MRS was used as a reference to measure Glu concentration in two different voxels, and this input was injected into the model to reduce the number of free parameters. Although ^1^H‐MRS became recognized as a standard technique for metabolic monitoring in the brain, quantitative ^1^H‐MRS is not straightforward. Proper quantification includes fitting of ^1^H‐MRS spectrum, then scaling results using a separately acquired water spectrum, and adding T_2_ and partial volume corrections.^32^ Few studies of quantitative ^1^H‐MRS in the mouse brain at 11.7 T have been published in literature and metabolite concentrations reported in these studies exhibited large variation. To accurately determine [Glu] in vivo, a whole ^1^H‐MRS pipeline was developed, including T_2_ measurement and partial volume correction based on brain tissues segmentation.

Finally, using the ^1^H‐MRS‐optimized multi‐pool model, the feasibility of quantitative gluCEST imaging was evaluated in vivo in the mouse brain, and the quality of the model was analyzed using simulations.

## 2 METHODS

### 2.1 Animal experiments

All experiments involving animals were approved by the local ethics committee and were performed according to French regulations (APAFIS no. 21 335‐201 907 031 642 584).

Seven‐ to 15‐month‐old wild type C57BL6 mice (Charles River) were anesthetized with isoflurane (1 to 1.5% isoflurane kept constant) in a 1:1 mixture of air and oxygen and were positioned on stereotaxic bed. Mouse temperature (37°C) and respiratory rate were monitored using PC SAM software (Small Animal Instruments) during scanning.

### 2.2 MRI acquisitions

All experiments were performed on a horizontal 11.7 T MRI scanner (Bruker).

#### 2.2.1 ^1^H‐MRS acquisitions

Localized ^1^H‐MRS spectra were acquired with a LASER (Localization by Adiabatic Selective Refocusing) sequence^33^ using a quadrature surface cryoprobe (Bruker) to increase SNR. A metabolite spectrum and an unsuppressed water spectrum were measured successively in the voxel of interest (VOI) to obtain quantitative measurements. For metabolite spectra acquisitions, a VAPOR^34^ (variable pulse power and optimized relaxa‐tion delays) water suppression module was used.

**FIGURE 1:**
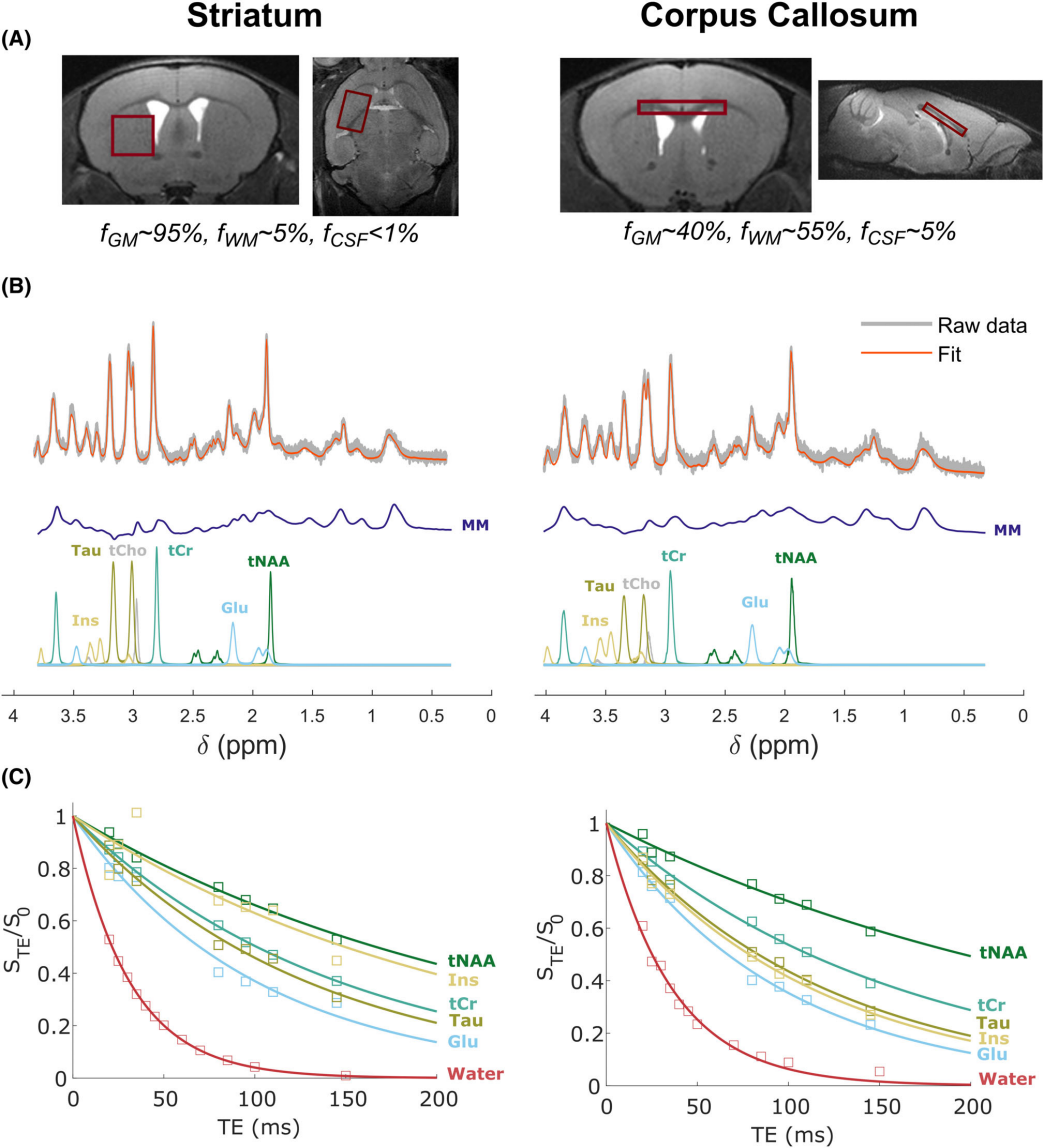
Quantitative ^1^H‐MRS acquisitions in the mouse brain. (A) Positioning of voxels of interest in striatum and corpus callosum. Typical fractions of gray matter, white matter, and CSF in the voxel are indicated. (B) Example of fit using LCModel toolbox of ^1^H‐MRS spectra acquired on two different animals at TE = 20 ms. Macromolecules (MM) spectrum was experimentally acquired, while metabolite spectra were simulated. (C) Typical plots of normalized metabolite concentrations fitted by LCModel and area under water peak as a function of TE. Solid lines indicate mono‐exponential fits for T_2_ estimation.

Two VOIs were chosen to investigate different tissue types (Figure 1A). The first VOI was placed in the striatum region, with a 12.8 μL volume (3.2 × 2 × 2 mm^3^), and contained almost exclusively gray matter. The second VOI was placed over the corpus callosum to target white matter tissue, with an 8 μL volume (4 × 0.5 × 0.5 mm^3^).

Metabolite and water ^1^H‐MRS spectra (32 and 16 averages, respectively) were acquired for quantification at TE = 20 ms, TR = 6 s. To estimate metabolite T_2_ values (in particular Glu's) in each VOI, additional metabolite spectra were also acquired at different TE, TE = [20, 25, 35, 80, 95, 110, 145] ms, specifically chosen to maximize Glu's signal, To estimate water's T_2_, spectra were acquired at TE = [20, 25, 30, 35, 40, 45, 50, 60, 70, 85, 100, 140, 200] ms. Experimental macromolecules (MM) spectra (4 × 64 averages) were acquired in each VOI on three animals with a double inversion‐recovery sequence (TI = 2200/770 ms, TR = 4050 ms) at all TE to incorporate the corresponding MM spectrum in LCModel^35^ basis‐set.

#### 2.2.2 Localized CEST measurements

Z‐spectra were acquired immediately after quantitative ^1^H‐MRS acquisitions in the same VOIs of the mouse brain. Localized Z‐spectra (Z‐spectra_localized_) were obtained using a spectroscopic approach^36^ (TE = 25 ms, TR = 5 s), based on the same LASER localization module preceded by a CEST saturation scheme. The CEST module consisted of a 1 s frequency‐selective quasi‐continuous wave pulse (10 × 100 ms rectangular pulses, separated by a delay of t_d_ = 10 μs). Fifty‐one saturation offsets were acquired after a dummy scan, ranging from −5 to +5 ppm by steps of 0.2 ppm with a saturation power of B_1_ = 5 μT. M_0_ acquired at δ = −100 ppm was used for Z‐spectrum normalization.

#### 2.2.3 CEST imaging of the mouse brain

CEST imaging was performed using a 72 mm quadrature volume coil (TX, Bruker) to yield an homogeneous B_1_ saturation in the whole brain, combined with a mouse‐head surface probe (RX, Bruker). Anatomical T_2_ images were acquired using the Bruker‐implemented multi‐spin‐multi‐echo sequence (0.1 × 0.1 mm^2^ resolution). CEST images were acquired using a rapid acquisition with relaxation enhancement (RARE) sequence (TR = 5 s, RARE factor = 10, TE = 30 ms) to image two slices of 0.6 mm of the mouse brain separated by 2.4 mm (0.15 × 0.15 mm^2^ resolution, 19.2 × 19.2 mm^2^ FOV, 480 mm^3^ shim volume). The same CEST scheme (t_sat_ = 1 s, B_1_ = 5 μT), was used to acquire the same 51 offsets between ±5 ppm and M_0_ at −100 ppm. For MTR_asym_ computation, B_0_ inhomogeneity was corrected using a water saturation shift referencing method^37^ acquired with B_1_ = 0.2 μT and offsets between −1 to +1 ppm by step of 0.1 ppm. These acquisitions typically took 1 h. Z‐spectra derived from CEST images are referenced as Z‐spectra_imaging_ throughout the manuscript.

### 2.3 Post processing

#### 2.3.1 Quantification of ^1^H‐MRS results

^1^H‐MRS spectra were fitted using LCModel toolbox.^35^ Quantification was achieved according to the experts‐recommended methodology,^32^ which is scaling by the unsuppressed water spectrum, along with T_2_ corrections and assessment of water concentration in the VOI through manual segmentation of tissues.^38^ Methodology is more thoroughly detailed in Appendix S1. This ^1^H‐MRS quantification pipeline was tested and validated in vitro (Figure S2).

#### 2.3.2 Correction of CSF contamination of the Z‐spectra_localized_

**FIGURE 2:**
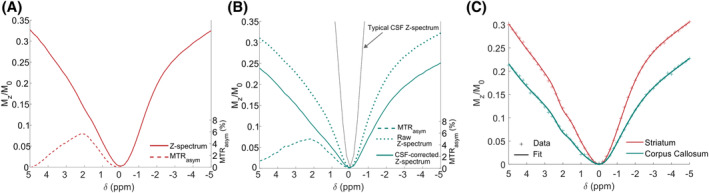
Mean Z‐spectra_localized_ measurements at B_1_ = 5 μT in the striatum and in the corpus callosum. (A) Average Z‐spectrum_localized_ measured at B_1_ = 5 μT in the striatum of five mice and the measurement of the magnetization transfer ratio with asymmetric analysis (MTR_asym_) (dashed line). (B) Average raw Z‐spectrum_localized_ (dashed fine line) and CSF‐corrected Z‐spectrum_localized_ (solid line) at B_1_ = 5 μT in the corpus callosum of seven mice. The gray line indicates the typical CSF Z‐spectrum used as reference for correction, and MTR_asym_ calculated on the corrected Z‐spectrum in bold dashed line. (C) Typical fit of Z‐spectra_localized_ acquired in striatum and corpus callosum of the same mouse using the optimized multi‐pool model.

Although the voxel positioned in the mouse striatum was carefully placed to avoid the contamination by ventricular signal, the inclusion of CSF could not be avoided in the case of the corpus callosum voxel, amounting to an estimated volumic fraction f_CSF_ = 5%–10% of the volume of tissues. To correct for the CSF contamination of the Z‐spectra_localized_ measured in the corpus callosum, a typical CSF Z‐spectrum *Z* ^*CSF*^ was acquired by averaging the CEST in ventricular areas (Figure 2B). The ratio of initial magnetization between the tissue of interest in the corpus callosum $M_{0}^{\mathrm{CC}}$and the CSF contribution $M_{0}^{\mathrm{CSF}}$ can be accounted for by water concentration difference and T_2_ effects:

(1) M0CSFM0CC=fCSF1−fCSF×[H2O]CSF[H2O]CC×exp−TET2CCexp−TET2CSF,

with a typical $T_{2}^{\mathrm{CSF}}$ of 110 ms (experimentally estimated). The experimentally measured signal is $M_{0}^{\exp}=M_{0}^{\mathrm{CC}}+M_{0}^{\mathrm{CSF}}$. Using the relation above, $M_{0}^{\mathrm{CC}}$ and $M_{0}^{\mathrm{CSF}}$ were estimated separately. From this, the corrected Z‐spectrum $Z_{\text{corr}}$ was derived from the experimentally acquired signal $M_{z}^{\exp}$:

(2) $$Z_{\text{corr}}=\frac{M_{z}^{\exp}-Z^{\mathrm{CSF}}M_{0}^{\mathrm{CSF}}}{M_{0}^{\mathrm{CC}}}.$$

### 2.4 CEST modeling methodology

First, the k_ex_^Glu^ was evaluated by fitting Z‐spectra_localized_ data with [Glu] fixed to ^1^H‐MRS measured value for each mouse and then averaged across all mice for both striatum (n = 5) and corpus callosum (n = 8). The k_ex_^Glu^ was fixed in the fitting model and [Glu] was left as a free parameter to be estimated on Z‐spectra_localized_ and Z‐spectra_imaging_ data.

#### 2.4.1 Z‐spectra_localized_ modeling

Z‐spectra_localized_ were fitted separately, with lower and upper bounds imposed on free variables with analytical Bloch‐McConnell simulations,^39^, ^40^ to save computing time, and using a least‐squares curve‐fitting algorithm (MATLAB 2022b). For each fit, residuals, R^2^, confidence intervals on fitted values at 95%, Akaike information criterion (AIC), corrected AIC (cAIC), and Bayesian information criterion (BIC) were computed (Appendix S1).

Several proton‐exchanging pools were included in the CEST models tested. MT was modeled with a super‐Lorentzian lineshape.^41^ MT pool has been reported to resonate at a negative frequency at low saturation power,^42^ so its resonance frequency was left as a free parameter between −2.5 and 0 ppm. To avoid unrealistic infinite absorption, the super‐Lorentzian lineshape was interpolated ±1 ppm around its resonance. To keep a reasonable number of free parameters, the resonance frequency of each pool was fixed to a known value (except for MT pool), the T_2_ value of all metabolites was set to 10 ms (except for MT pool where it was fixed to 10 μs). The same T_1_ was used for all pools (i.e., T_1_ of water). During the fit, freedom was left in a range of plausible variation around the measured values in each ROI for T_1_ and T_2_ of water, which allows adjusting the direct water saturation width.

#### 2.4.2 Mapping of Glu concentration using CEST modeling

CEST images were denoised on k‐space data using the multilinear singular value decomposition method^43^ (core size of 30 × 30 for spatial dimensions and 10 for spectral dimension). Rician noise was corrected by removing and rescaling by non‐zero CEST signal ˜ 0 ppm.^44^

Z‐specta_imaging_ were then fitted pixel‐by‐pixel in the brain region using the determined quantitative model described in Table S1. For each slice, a manually segmented mask of corpus callosum was used to differentiate the fit parameters used in gray matter and white matter.

From fitted Glu‐proton fractions, [Glu] maps were estimated with:

(3) $$\left[\mathrm{Glu}\right]=\frac{f_{H}^{\mathrm{Glu}}}{3}\times 2\left[H2O\right],$$

with [H_2_O] = 40.0 M in corpus callosum and [H_2_O] = 46.1 M in the rest of the brain.^38^

## 3 RESULTS

### 3.1 Quantitative ^1^H‐MRS

LCModel analysis provided a reliable fit with Cramér–Rao bound <5% in both voxels (Figure 1B), for Glu, total creatine (tCr), myo‐inositol (Ins), total N‐acetyl aspartate (tNAA), and taurine (Tau).

T_2_ values of the main metabolites were estimated with monoexponential fits (Figure 1C) and averaged over five animals (Table 1). Note that our set and range of TE values were chosen as a compromise to maximize Glu signal when accounting for J‐couplings, and therefore, are not the best for the estimation of other metabolites' T_2_. Even so, T_2_ fits for Glu were not optimal, with an average R^2^ of 0.97. This subpar quality of T_2_ fit was likely because of inaccurate simulation of how J‐couplings affect the MRS signal, especially at longer TE, because of potentially slightly inaccurate J values. The same was also observed for Ins (R^2^ ≈ 0.96), but not for metabolites without J‐couplings, like tCr, or tNAA (R^2^ > 0.98). This difficulty has been reported before for metabolites with strong J‐couplings.^38^, ^45^ Moreover, Glu T_2_ estimation would be improved if more points were acquired at longer TE, but this proved impossible to do because Glu signal rapidly drops at TE >150 ms. Nonetheless, T_2_ estimations for water were reliable with R^2^ >0.99, which is the most primordial for quantitative spectroscopy.

**TABLE 1:** ^1^H‐MRS results in the mouse brain: T_2_ values and metabolite concentrations.

	Glu	tCr	Ins	tNAA	Tau	Water
Measured T_2_ (ms)						
Striatum	107 ± 9	145 ± 12	188 ± 19	243 ± 19	125 ± 9	31.7 ± 0.6
Corpus callosum	99 ± 8	159 ± 4	129 ± 10	278 ± 20	125 ± 6	28.8 ± 1.2
Concentration in VOI (mM)						
Striatum	6.5 ± 0.3	7.2 ± 0.2	3.4 ± 0.3	5.3 ± 0.1	12.2 ± 0.7	45.5 ± 0.2
Corpus callosum	5.7 ± 0.4	5.8 ± 0.2	3.3 ± 0.2	4.6 ± 0.3	8.0 ± 0.2	46.5 ± 0.7 M

*Note*: T_2_ values were averaged on five mice. Concentrations were averaged on 10 mice. SDs between animals are provided.

Abbreviations: Glu, glutamate; Ins, myo‐inositol; tCr, total creatine; tNAA, total N‐acetyl aspartate; Tau, taurine; VOI, voxel of interest.

Estimated concentrations of main metabolites are reported in Table 1, averaged over 10 animals. Glu concentration measured in the striatum and the corpus callosum were 6.5 ± 0.3 mM and 5.7 ± 0.4 mM, respectively. Moreover, four successive measurements were made in the same experiment on five animals to ensure that [Glu] estimations were repeatable: mean intra‐individual SD were estimated to be 0.25 mM and 0.30 mM, respectively, in striatum and in corpus callosum.

Overall, concentrations of [Glu] were estimated to be of 5 to 7 mM using quantitative ^1^H‐MRS, which is slightly lower than what has been reported in literature in the mouse brain (6–10 mM).^46^, ^47^, ^48^, ^49^ It is known that concentrations evaluated through quantitative spectroscopy can be potentially biased by a systemic factor, due for instance to hardware, erroneous T_2_ values, or to fitting bias.^32^, ^50^, ^51^ However, our quantitative ^1^H‐MRS pipeline was validated on phantoms (Figure S2), where error on [Glu] was of 8% in average, ensuring that there was no significant bias because of hardware.

### 3.2 CEST modeling in vivo

#### 3.2.1 Optimization of the multi‐pool model

Many biological molecules are known to exhibit a CEST signal,^19^ and therefore, might contribute to the Z‐spectrum measured in vivo at 5 μT. However, to develop a robust quantitative model, the number of free parameters must be limited, and including too many proton‐exchanging pools should be avoided, because it might lead to overfitting. At lower saturation powers, CEST signatures of pools like amides, Cr or NOE come out on the Z‐spectrum as clearly defined peaks,^31^, ^52^ but at 5 μT, no clear resonance of a particular pool can be distinguished on the MTR_asym_ (Figure 2). Hence, we chose in this work to empirically determine the best combination of CEST pools that would give the optimal quality of fit of our data. Different combinations of typical proton‐exchanging pools to include in our model were tested, and AIC, cAIC, and BIC were computed and minimized to find the best model to fit our data without overfitting.

Several candidate proton‐exchanging pools aside from MT were considered to model the Z‐spectrum_localized_ at 5 μT. Fixed and free parameters of the different proton‐exchanging pools were defined in Tables 2 and 3 respectively:

- Glu was modeled as a 3‐protons exchanging group resonating at 3.0 ppm. In an effort to reduce the number of unknown parameters, [Glu] was fixed in this optimization step to the value found with quantitative spectroscopy measurements, whereas k_ex_^Glu^ was left as a free parameter.
- A guanidinium (Guan) pool, resonating at 2.0 ppm, with an exchange rate fixed at 300 Hz, which is approximately the value which has been reported in the mouse brain for Cr.^31^
- An amide (APT) pool, resonating at 3.5 ppm, with an exchange rate fixed at 22 Hz, as already reported in the rodent brain.^52^, ^53^, ^54^
- An hydroxyl (OH) pool, resonating at 1.0 ppm. The exchange rate was fixed at 2000 Hz, as reported in vitro for Ins.^19^
- Two NOE pools, resonating, respectively, at −3.5 ppm and − 1.6 ppm. Although the first NOE^1^ pool has been extensively studied, with a typical exchange rate of 15 Hz,^52^ the existence of the second NOE^2^ pool was demonstrated only recently.^55^ Its exchange rate has never been quantified to our knowledge, but the optimal saturation power observed to maximize the signal of this pool^56^ hints at an exchange rate of the order of 10 Hz, which is the value we fixed here.
- A Tau pool resonating at 3.2 ppm. Although the presence of Tau‐related CEST signal has never been demonstrated in vivo, in vitro studies have demonstrated a fast‐exchanging proton dynamic,^19^, ^29^ which therefore, make Tau a potential candidate to contribute to CEST signal at 5 μT, especially considering [Tau] is ˜10 to 15 mM in the mouse brain. An exchange rate of 50 000 Hz was used^19^ (lower value of 10^4^ Hz^29^ was also tried in Table S4).

**FIGURE 3:**
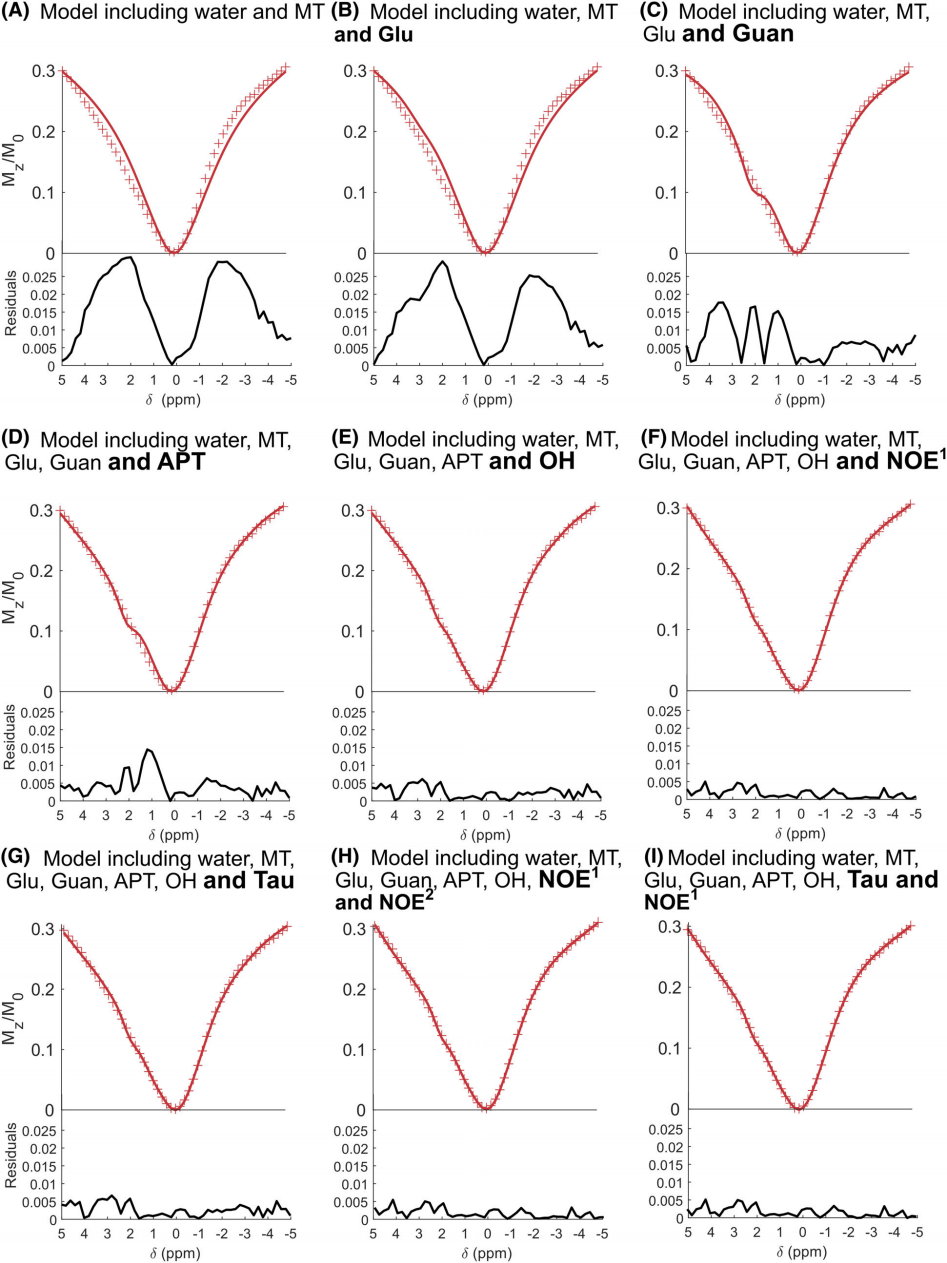
Selection of a suitable multi‐pool model for glutamate‐weighted CEST (gluCEST) modeling. Modeling of Z‐spectra_localized_ data with several models including different proton‐exchanging pools, with [Glu] fixed to the ^1^H‐MRS m easured value. Typical fits (solid line) of experimental data points (red crosses) acquired in the striatum of a mouse at B_1_ = 5 μT using several models. Residuals (absolute values) are plotted under each fit.

Figure 3 shows several fits of Z‐spectra_localized_ acquired in vivo in the striatum with different combinations of pools. Criterions of fit quality are reported in Table 4. Additional combinations were tested in Table S4. The best model, with minimum AIC, cAIC, and BIC, included MT, Glu, Guan, APT, OH, and NOE^1^ as proton‐exchanging pools. Moreover, the evolution of residuals (Figure 3) when including these pools in the model further validates their relevance. For instance, the addition of the Guan pool (Figure 3C) significantly decreased the peak of residuals at 2.0 ppm. Similarly, the inclusions of APT pool (Figure 3D), OH pool (Figure 3E), or NOE^1^ (Figure 3F) pool reduced respectively the residual peaks at 3.5 ppm, 1.0 ppm, and in the negative offsets. However, at this saturation power, addition of Tau and NOE^2^ pools systematically increased AIC and BIC, and therefore, were not taken into account in the final model.

**TABLE 2:** Fixed parameters of the different proton‐exchanging pools tested in the fitting models of CEST data in Figure 3.

Fixed parameters of the candidate pools		
Pool	Parameter	Fixed value
Glu	δ^Glu^	3.0 ppm
	f^**Glu**^	Fixed with MRS
Guan	δ^Guan^	2.0 ppm
	k_ex_^Guan^	300 Hz
APT	δ^APT^	3.5 ppm
	k_ex_^APT^	25 Hz
OH	δ^OH^	1.0 ppm
	k_ex_^OH^	2000 Hz
NOE^1^	δ^NOE1^	−3.5 ppm
	k_ex_^NOE1^	15 Hz
NOE^2^	δ^NOE2^	−1.6 ppm
	k_ex_^NOE2^	10 Hz
Tau	δ^Tau^	3.2 ppm
	k_ex_^Tau^	50 000 Hz

*Note*: All T_2_ values for metabolites were set to 10 ms, and to 10 μs for MT pool. T_1_ values were set to the same value as water pool.

Abbreviations: APT, amide; Glu, glutamate; Guan, guanidium; OH, hydroxyl; NOE, nuclear Overhauser effects; Tau, taurine; k_ex_^*i*^, exchange rate of pool *i*.

**TABLE 3:** Free parameters of the different proton‐exchanging pools tested in the fitting models of CEST data in Figure 3.

Free parameters of the candidate pools				
Pool	Parameter	Initial value	Lower bound	Upper bound
Water	T_1_	1.95 s (striatum) 1.85 s (corpus callosum)	−10%	+10%
	T_2_	31.7 ms (striatum) 28.8 ms (corpus callosum)	−10%	+10%
MT	δ^MT^	−2.34 ppm	−2.5 ppm	0 ppm
	k_ex_^MT^	20 Hz	10 Hz	60 Hz
	f_H_^MT^	5%	0%	50%
Glu	k_ex_^Glu^	8000 Hz	0 Hz	20 000 Hz
	f_H_^Glu^	Fixed with MRS	Fixed with MRS	Fixed with MRS
Guan	f_H_^Guan^	0.07%	0%	2.2%
APT	f_H_^APT^	0.2%	0%	10%
OH	f_H_^OH^	0.03%	0%	0.6%
NOE^1^	f_H_^NOE1^	0.5%	0%	8%
NOE^2^	f_H_^NOE2^	0.5%	0%	8%
Tau	f_H_^Tau^	0.03%	0%	0.3%

*Note*: All T_2_ values for metabolites were set to 10 ms, and to 10 μs for MT pool. T_1_ values were set to the same value as water pool.

Abbreviations: APT, amide; Glu, glutamate; Guan, guanidium; MT, magnetization transfer; OH, hydroxyl; NOE, nuclear Overhauser effects; Tau, taurine; k_ex_^*i*^, exchange rate of pool *i*; f_H_^*i*^, proton fraction of pool *i*.

**TABLE 4:** Suitability of the candidate models for fitting Z‐spectra_localized_ data.

Pools included in the candidate model	R^2^	AIC	cAIC	BIC
Water, MT (Figure 3A)	0.96211	−250	−248	−240
Water, MT, Glu (Figure 3B)	0.97140	−262	−260	−250
Water, MT, Glu, Guan (Figure 3C)	0.99225	−327	−324	−313
Water, MT, Glu, Guan, APT (Figure 3D)	0.99709	−375	−371	−359
Water, MT, Glu, Guan, APT, OH (Figure 3E)	0.99912	−433	−429	−416
Water, MT, Glu, Guan, APT, OH, NOE^1^ (Figure 3F)	0.99958	−469	−464	−450
Water, MT, Glu, Guan, APT, OH, Tau (Figure 3G)	0.99912	−431	−426	−412
Water, MT, Glu, Guan, APT, OH, NOE,^1^ NOE^2^ (Figure 3H)	0.99958	−467	−460	−446
Water, MT, Glu, Guan, APT, OH, NOE,^1^ Tau (Figure 3I)	0.99957	−466	−459	−444

*Note*: Quality of each model using several statistical criterions. Best quality is achieved for the highlighted model. Additional combinations are reported in the Table S3.

Abbreviations: AIC, Akaike information criterion; cAIC, corrected AIC; APT, amide; BIC, Bayesian information criterion; Glu, glutamate; Guan, guanidium; Ins, myo‐inositol; MT, magnetization transfer; NOE, nuclear Overhauser effects; OH, hydroxyl; tCr, total creatine; tNAA, total N‐acetyl aspartate; Tau, taurine; VOI, voxel of interest.

#### 3.2.2 Glu protons exchange rate in vivo

To obtain accurate [Glu] quantification for Glu mapping, it is essential to first estimate the exchange rate of its amine protons in vivo. Using the optimized multi‐pools model, the set of Z‐spectra_localized_ was fitted with prior fixed value on [Glu] thanks to individual ^1^H‐MRS measurements performed in the same VOI, in both striatum and corpus callosum (Figure 2C and Table 5). Good quality of fits were obtained, with mean R^2^ of 0.9996 in striatum, and a bit lower quality of R^2^ of 0.996 in the corpus callosum, probably because of noisier data. Glu exchange rate was estimated to be 1301 ± 21 Hz (n = 5 mice) with a pretty good confidence (average 95% CI of 19 Hz) in the striatum. In the corpus callosum, Glu exchange rate was estimated with much more variability (k_ex_^Glu^ = 1276 ± 76 Hz, n = 8 mice), probably because of the difficulty to place the VOI reproducibly between animals in the corpus callosum, and with less confidence because of noise (average 95% CI of 161 Hz).

**TABLE 5:** Average fitting results in the striatum and corpus callosum and SDs.

Mean R^2^	Striatum		Corpus callosum	
	0.9996		0.9959	
	Mean fitted value	Mean 95% CI	Mean fitted value	Mean 95% CI
k_ex_^Glu^	1301 ± 26 Hz	19 ± 9	1276 ± 76 Hz	161 ± 151
f_H_^Guan^	0.4 ± 0.1%	(2 ± 1)e–3%	0.7 ± 0.2%	(17 ± 15)e–3%
f_H_^APT^	3.5 ± 0.5%	(14 ± 6)e–3%	4.4 ± 0.8%	(109 ± 105)e–3%
f_H_^OH^	0.5 ± 0.3%	(2 ± 2)e–3%	0.5 ± 0.6%	(6 ± 4)e–3%
f_H_^NOE^	4.1 ± 0.2%	(34 ± 9)e–3%	3.6 ± 1.2%	(181 ± 206)e–3%
f_H_^MT^	10.4 ± 0.9%	0.11 ± 0.04%	12.9 ± 5.6%	0.8 ± 1.3%
k_ex_^MT^	13.3 ± 0.8 Hz	0.20 ± 0.05 Hz	25.8 ± 18.9 Hz	0.16 ± 0.25 Hz
δ^MT^	−0.03 ± 0.04 ppm	0.04 ± 0.01 ppm	−0.53 ± 0.82 ppm	0.2 ± 0.25 ppm

*Note*: Fitted values were averaged on 5 mice for the striatum and 8 mice for the corpus callosum.

Abbreviations: APT, amide; Glu, glutamate; Guan, guanidium; MT, magnetization transfer; OH, hydroxyl; NOE, nuclear Overhauser effects; Tau, taurine.

#### 3.2.3 Quantitative Glu mapping in the mouse brain

**FIGURE 4:**
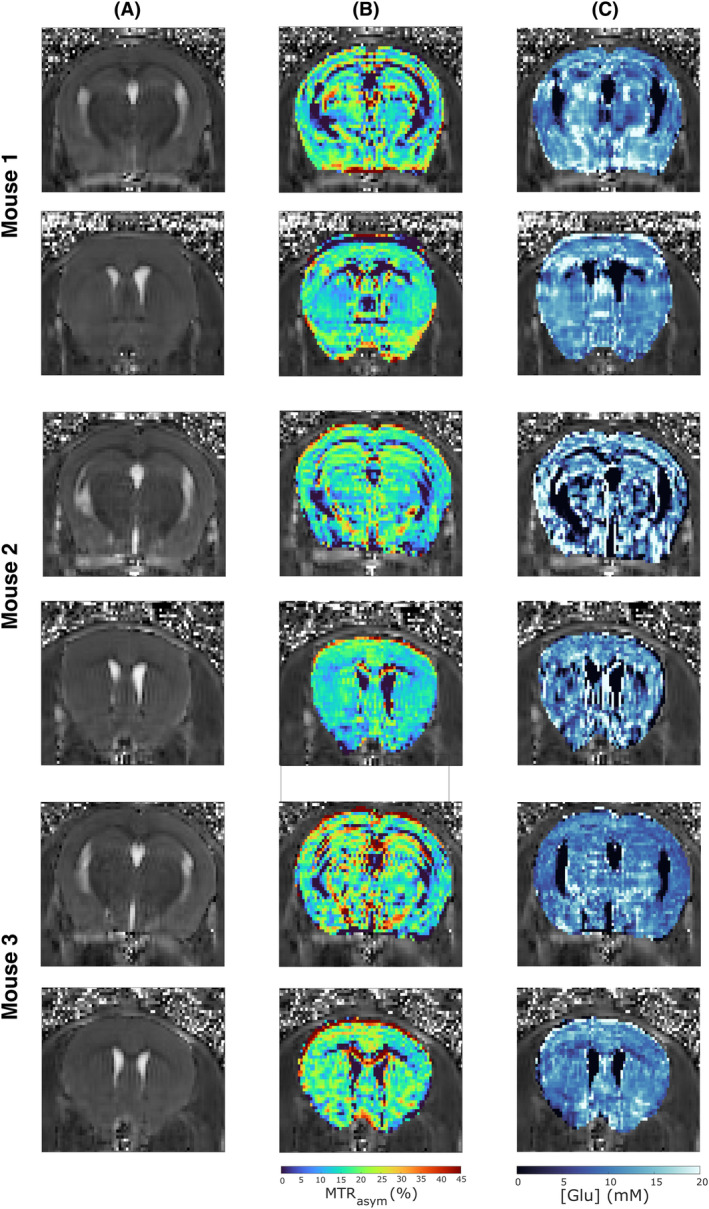
Quantitative glutamate mapping in the mouse brain in several mice. (A) M_0_ images acquired at −100 ppm and (B) measurement of the magnetization transfer ratio with asymmetric analysis (MTR_asym_) (3 ppm) maps corrected with the water saturation shift referencing and normalized by M_Z_(−3 ppm).^1^ (C) [Glu] maps obtained by pixel‐by‐pixel fit of Z‐spectra_imaging_ data acquired at B_1_ = 5 μT.

Using the optimized multi‐pool model and the estimated value of Glu's exchange rate, CEST images were acquired in the mouse brain to fit pixel by pixel the corresponding Z‐spectrum_imaging_. Table S1 reports the parameters and bounds used for fitting. Representative [Glu] maps obtained in three different mice are shown in Figure 4C.

Average [Glu] values were evaluated in several manually segmented regions of the mouse brain (Figure S3). On average, [Glu] = 9.1 ± 0.6 mM was estimated in the corpus callosum, 11.8 ± 1.0 mM in cortex, 11.2 ± 1.6 mM in hippocampus and 10.9 ± 0.8 mM in striatum. By calculating average ratio of mean to SD in those regions, a better SNR was systematically observed in quantitative [Glu] maps (1.95, 3.98, 4.32, and 5.01, respectively) than in conventional MTR_asym_ contrast maps (0.57, 3.10, 3.57, and 3.96, respectively). Lower [Glu] were systematically found in corpus callosum than in other regions. Ventricular regions, with almost zero [Glu], were more clearly defined on [Glu] maps (Figure 4C) compared to MTR_asym_(3 ppm) maps (Figure 4B).

#### 3.2.4 Quality of the quantitative fitting model

To assess the quality of the fitting model, several sets of Z‐spectra at 5 μT were simulated and fitted with our quantitative pipeline. In each simulation, T_1_, T_2_, [Glu], [Guan], [OH], [NOE], f^H^_MT_, k_ex_^MT^, and ΔB_0_ were varied in a range of realistic values (Table S5). The accuracy and precision of our model was estimated on a set of 2000 simulations, with different levels of Rician noise.

**FIGURE 5:**
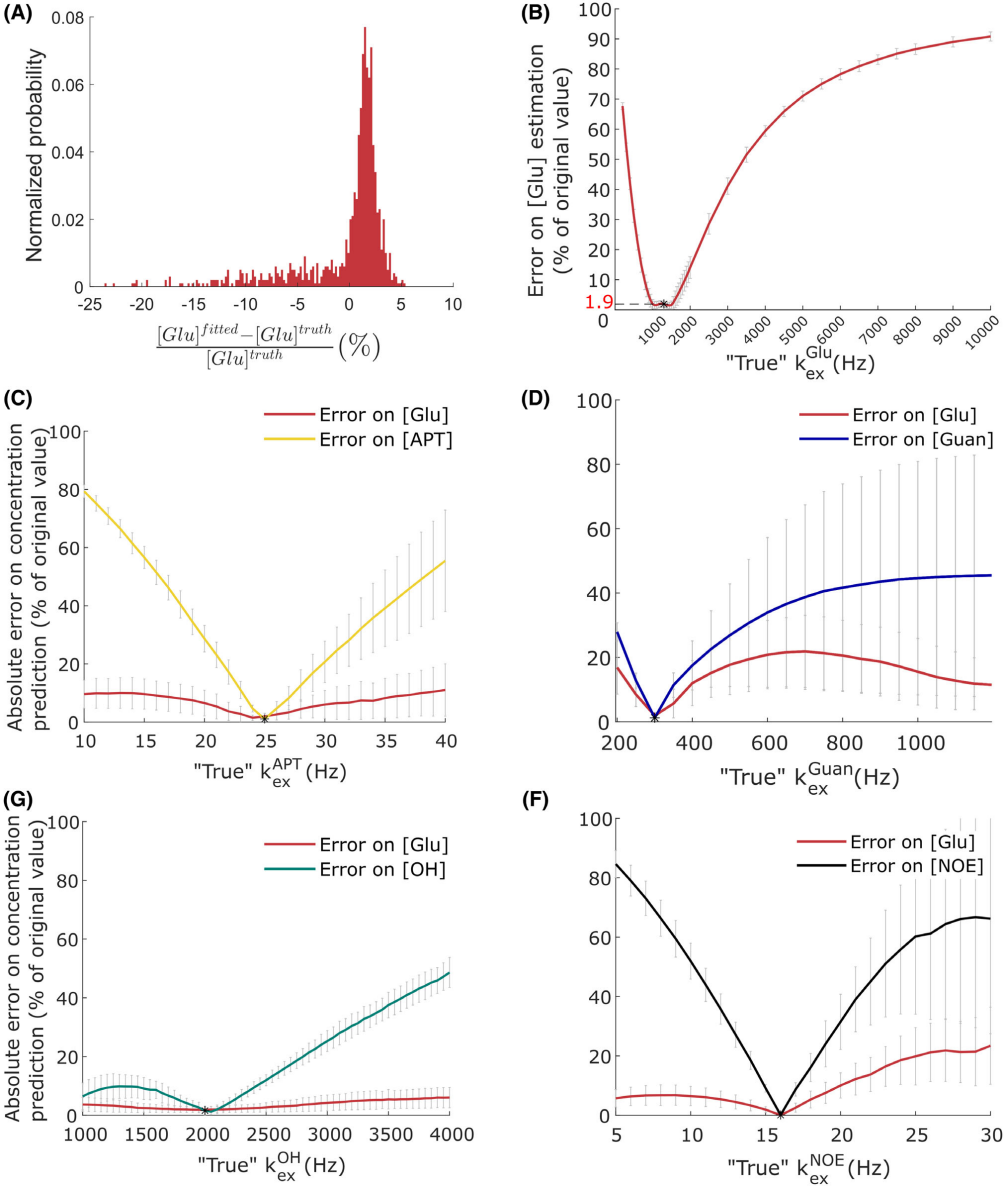
Accuracy, precision and robustness of the fitting model. (A) Histogram of estimation errors of the fitting model on a set of 2000 simulations (no noise). (B–F) Robustness of the fitting model to deviations in exchange rate values varying “true values” of exchange rate k_ex_^Glu^, k_ex_^APT^, k_ex_^Guan^, k_ex_^OH^, k_ex_^NOE^ respectively, with additionally the mean absolute error made on estimations of [APT], [Guan], [OH] and [NOE], respectively.

On average, without noise (Figure 5A), the model has intrinsic good accuracy ([Glu] error limited to 1.4%), and good precision (SD of 1.4%). However, when noise is added to the data, the precision degrades quickly, with SD of error rising up to 152% for 2% of Rician noise (Table S6). Accuracy remains good for low level of noise. When noise rises above 1%, the fitting algorithm tends to overestimate [Glu], up to +41% for 2% of noise. This highlights the sensitivity of the model to noise and the need to use denoised CEST data for fitting.

Additionally, the robustness of the quantitative model to potential deviations in exchange rate values was evaluated. Indeed, physiological changes (pH, temperature, …) may impact exchange rates and could lead to biased estimation of [Glu]. Multiple sets of 100 simulations with different values of pools' exchange rates were generated and then fitted to estimate the absolute error of the quantitative model. The error made on [Glu] estimations remained acceptably low (< 20%) when k_ex_^Guan^, k_ex_^APT^, k_ex_^OH^, or k_ex_^NOE^ deviate from the values hypothesized in the model (Figure 5C–F). However, [Glu] estimations were significantly more sensitive to potential deviations in k_ex_^Glu^ (Figure 5B). For instance, if the exchange rate of Glu was 3500 Hz instead of the previously determined 1301 Hz, then the typical [Glu] error could rise to 50%. Nonetheless, the accuracy remained rather good, with an error <10%, for a k_ex_^Glu^ ranging from 800 to 1850 Hz.

## 4 DISCUSSION

We developed a multi‐pool CEST model to quantify Glu concentration in vivo and to establish quantitative maps in the mouse brain. The choice of using ^1^H‐MRS as an internal reference was motivated by the need to have an in vivo control of [Glu] to calibrate a CEST model, and because post‐mortem and in situ measurements of [Glu] are known to deviate from in vivo values.^57^

In this study, the relevant exchanging‐proton pools to include in the model to describe the CEST signal at 5 μT were empirically determined. A 6‐pool model including MT, Glu, Guan, APT, OH, and NOE (−3.5 ppm) matched data the best (Figure 3) without overfitting (Table 4). This empirically determined model is not necessarily the “true” model to describe the CEST signal at this saturation power, especially because only a few candidate pools that are usually found in literature were tested. Nevertheless, using this model, the typical contribution of Glu to the MTR_asym_ (3 ppm) at 5 μT is estimated to be ˜15% (Figure S4). However, based on typical concentrations and exchange rate values reported in vitro,^19^ a contribution of Glu of ˜30% would have been expected at 5 μT. This mismatch can be explained because in vitro measurement is not enough to describe in vivo data. Note that this estimation of 15% Glu contribution to gluCEST signal is close to the result of Cui et al.,^58^ which reported a Glu contribution <12% at 3.6 μT in the rat brain.

One of the main parameter influencing the [Glu] estimation is the value of the exchange rate of amine protons. One result of particular interest was the in vivo estimation of Glu exchange rate, measured at 1301 Hz and 1276 Hz in the striatum and in the corpus callosum respectively. These values are significantly lower than the typical values of 5000 to 9000 Hz that can be measured in vitro at pH = 7 (Figure S1 and Table S3, as well as values reported in the literature^1^, ^19^, ^29^, ^30^). This can be explained by the fact that the exchange dynamics of Glu's protons are extremely sensitive to its chemical environment: pH, but also the type of buffer (Table S3) and its concentration.^29^ Hence, it might not be unrealistic to have a lower k_ex_^Glu^ in vivo. Therefore, we hypothesize that the physiological environment of Glu in the mouse brain considerably slows down the exchange dynamics of its protons. Note that this conclusion is consistent with the work of Zhang et al.^31^ which found a 2 to 3 times lower exchange rate of Cr in the mouse brain as compared to in vitro studies. This result also points out that using Glu exchange rate determined ex vivo in a buffer, far from physiological environment, can drastically affect modeling of in vivo data and can lead to biased estimation of [Glu] (Figure 5B).

A limit of this quantification model is that it is not suitable to map other metabolite's concentrations, as it was designed to evaluate [Glu]. In particular, the exchange rates of these other pools (Guan, APT, OH, and NOE^1^) were fixed to values reported in literature, in an effort to reduce the number of free parameters and make [Glu] estimations more robust. These k_ex_ values were usually measured at lower saturation powers or in vitro, and might not the best to describe the CEST signal at 5 μT. For instance, suspicions of the existence of fast exchanging Guan pool^59^ or proteins^58^ (i.e., amides have been reported before). To describe these potential pools, the low k_ex_ values chosen here might not be appropriate. This most likely explains why fitted values of f_Guan_ and f_APT_ are so high in Table 5 (>100 mM for instance for a Guan pool with 4 protons per molecule). Although the k_ex_ values of these pools seem to not affect too much the [Glu] predictions of our model, predictions of [Guan], [APT], [OH], or [NOE^1^] are very sensitive to the first hypothesis on their exchange rate value (Figure 5C–F). Considering additionally that B_1_ = 5 μT is not an optimal saturation power for sensitive detection of these pools, we conclude that mapping of [Guan], [APT], or [OH] using the model developed here is unreliable.

**FIGURE 6:**
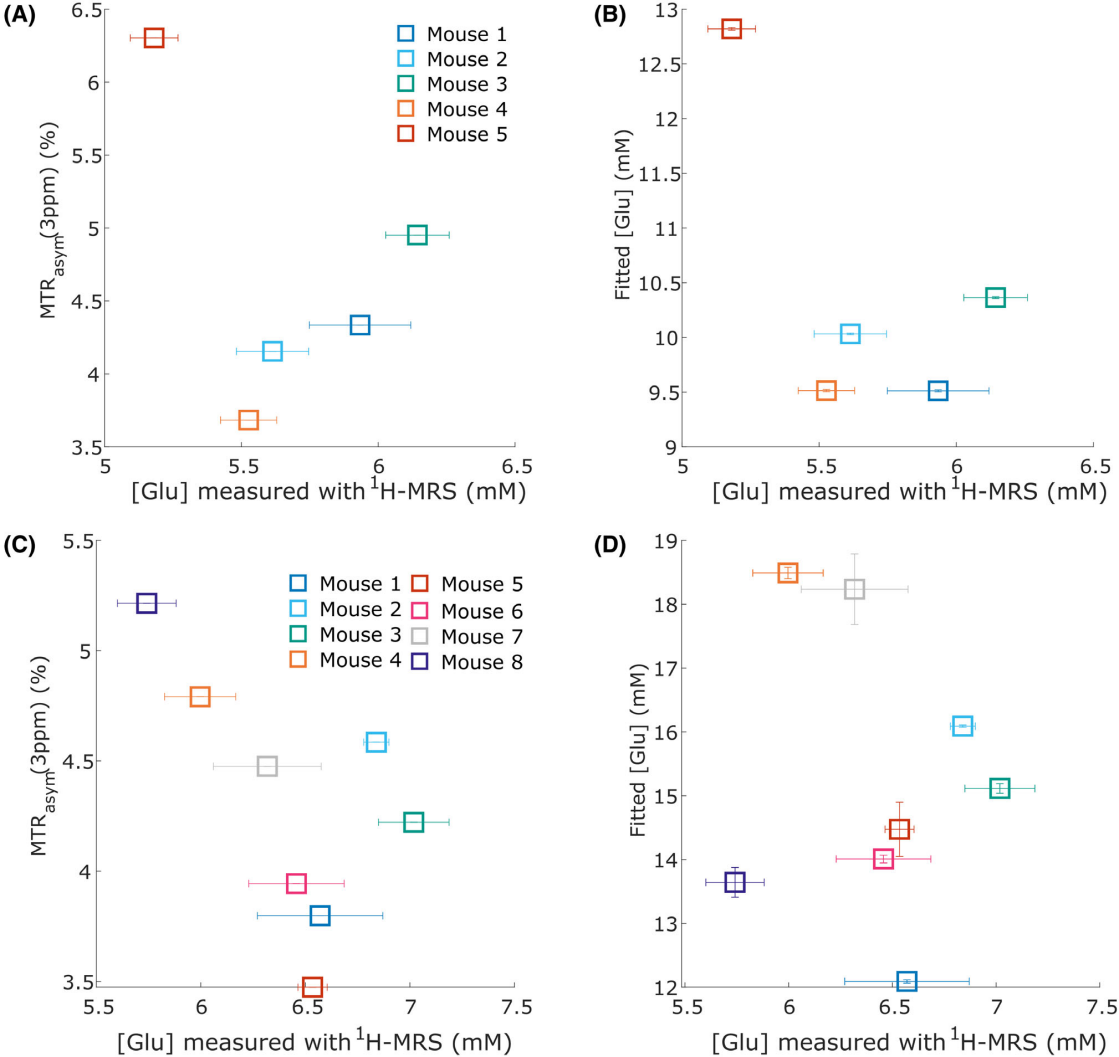
Prediction of [Glu] by quantitative CEST model versus ^1^H‐MRS measurements on localized LASER data. (A) Plot of the measurement of the magnetization transfer ratio with asymmetric analysis (MTR_asym_) (3 ppm) versus the measured [Glu] with ^1^H‐MRS for n = 5 mice in the striatum voxel of interest (VOI). Error bars indicated in the x‐axis direction correspond to the standard error of the mean estimated for individual ^1^H‐MRS measurements. (B) [Glu] predicted by the quantitative glutamate‐weighted CEST (gluCEST) fitting model of Table S1 versus the measured [Glu] with ^1^H‐MRS for n = 5 mice in the striatum VOI. On average, the predicted [Glu] was overestimated by 4.8 ± 1.6 mM. Error bars indicated in the y‐axis direction correspond to the 95% CI estimated for [Glu] predictions. (C) Plot of the measurement of the magnetization transfer ratio with asymmetric analysis (MTR_asym_) (3 ppm) versus the measured [Glu] with ^1^H‐MRS for n = 8 mice in the corpus callosum VOI. (D) [Glu] predicted by the quantitative gluCEST fitting model of Table S1 versus the measured [Glu] with ^1^H‐MRS for n = 8 mice in the corpus callosum VOI. On average, the predicted [Glu] was overestimated by 8.8 ± 2.3 mM.

Interestingly, the fitting model tended to predict higher [Glu] on experimental Z‐spectra_imaging_ data (in the range of 9–13 mM) (Figure 4C) than the typical [Glu] predicted by ^1^H‐MRS (˜5–7 mM) (Table 1). This inaccuracy in prediction cannot be explained by a fitting bias alone. We estimated in our experimental conditions that the noise level of our Z‐spectra_imaging_ data was ˜0.5%, which, according to Table S6, should not lead to overestimation. This effect was also observed when fitting Z‐spectra_localized_ data acquired in the striatum or corpus callosum (Figure 6), where predicted [Glu] was on average overestimated by, respectively, 4.8 mM and 8.8 mM, but with rather consistent bias (SD = 1.6 mM and 2.3 mM). It appears that relaxing the constraint on [Glu] in the fitting process inevitably leads to converging toward a higher [Glu] than that measured by ^1^H‐MRS, even with a properly defined k_ex_^Glu^. Therefore, one more likely explanation to this systematic overestimation of [Glu] might be the existence of another fast‐exchanging pool contributing to gluCEST signal, which was not considered here, because it has been previously suggested by Cui et al.^58^. In fact, we hypothesized that cellular Glu was the only proton‐exchanging pool accountable at 3.0 ppm. There might also be other molecules (like GABA or Tau) with amine groups resonating at 3.0 ppm, although the convincing correlations between [Glu] and MTR_asym_ (3 ppm) that have been reported before^5^, ^7^ hints at rebuking this hypothesis. Still, the existence of a second pool of Glu, encapsulated in synaptic vesicles, with different exchange dynamics, is an hypothesis that has been suggested before.^58^ In particular, this second pool of Glu might not be well detected by ^1^H‐MRS if its T_2_ were ultra‐short, which could impact the results of the modeling methodology presented in this article and could explain the discrepancy in Glu concentration estimated using quantitative gluCEST and quantitative ^1^H‐MRS approaches. Nevertheless, although the molecular origin of this potential unknown pool cannot be determined with certainty, the quantification bias of [Glu] appears to be systematic, which makes the fitting model still relevant.

Despite this point, this study is, to our knowledge, the first to propose a method to perform quantitative in vivo [Glu] mapping, with a much better resolution than what can be achieved with ^1^H‐MRS. Compared to the conventional gluCEST contrast, this quantitative [Glu] mapping allowed for more reliable identification of Glu repartition in several brain regions (Figure 4), with better SNR on quantitative maps and provides a more universal metric than MTR_asym_. However, the sensitivity of the quantification model to noise is also highlighted in Table S6, which points out the need for high SNR or efficient denoising techniques. Furthermore, at lower field strength, quantification of [Glu] is likely to lose in accuracy, because of the reduced spectral separation in CEST resonance frequencies. However, these flaws, along with the current long acquisition time (˜1 h compared to 20–30 min for conventional gluCEST imaging), might be overcome using machine learning or fingerprinting techniques to optimize acquisition point sampling.^60^

## 5 CONCLUSION

We propose in this work a quantitative gluCEST model and demonstrate its ability for high‐resolution Glu mapping in the mouse brain. In particular, we reported a much lower Glu exchange rate in the mouse brain than what can be measured in vitro. This highlights the need to develop quantitative gluCEST modeling while accounting for the differences between in vitro and in vivo conditions, which were largely achieved here by calibrating the fitting model using ^1^H‐MRS as an internal reference in vivo, although further investigations might be required about the contributions to gluCEST signal of other unknown in vivo CEST agents. This work, therefore, potentially opens new perspectives for neuroimaging and new possibilities for the monitoring of metabolism in the context of neuropathologies.

## Supporting information


**Appendix S1.** Supporting Information.

## Data Availability

The source codes and data underlying the results are available at https://osf.io/65n8c/.
