# Supplementary material for: Toward quantitative CEST imaging of glutamate in the mouse brain using a multi‐pool exchange model calibrated by 1H‐MRS
Source: Magn Reson Med. 2024 Oct 24;93(3):1394–410. doi: 10.1002/mrm.30353 (PMC11680732; doi:10.1002/mrm.30353)
Supplement: Supplementary file 1 — Appendix S1. Supporting Information. [file MRM-93-1394-s001.docx]

# **Supplementary material**

### **Appendix S1: Detailed quantitative ^1^H-MRS method**

Eddy-current correction was performed both for metabolite and water spectra, by removing from both FIDs the phase of the water FID:

$$\text{FID}_{\text{corr}}=\text{FID}_{\text{raw}}\times e^{-i\varphi_{water}\left( t \right)}$$

Metabolite spectra were fitted using the LCModel toolbox ^34^. A water scaling ratio was calculated by comparing, relatively to the number of protons, the integral of the water peak to the integral of a reference metabolite singlet (here the singlet of phosphocreatine at 3.03 ppm). Quantification relative to water was achieved by multiplying LCModel’s output values by this water scaling ratio and correcting T_2_ effects ^31^:

$$\text{f}\text{Metab}=\frac{\left[ \text{Metabolite} \right]\text{app}}{[\text{H}\text{2}\text{O}]}=\left( \frac{\left[ \text{Metabolite} \right]}{\left[ \text{PCr}\text{3ppm} \right]} \right)\text{LCModel}\times\frac{\frac{1}{3}\int\text{PCr}\text{3ppm}\text{ peak}}{\frac{1}{2}\int\text{H}\text{2}\text{O peak}}\times\frac{exp(-\frac{\text{TE}}{\text{T}\text{2}\text{H}\text{2}\text{O}})}{exp(-\frac{\text{TE}}{\text{T}\text{2}\text{Metab}})}$$

To reach absolute quantification, this quantity needs to be multiplied by the water concentration in the voxel. For pure water (phantom experiments), [H_2_O] = 55.6 M, but in the case of in vivo experiments, the water concentration depends on the tissue composition within the VOI. For each animal, manual segmentation of VOIs was performed on T_2_-weighted anatomical images to estimate fractions of grey matter (f_GM_), white matter (f_WM_) and cerebrospinal fluid (f_CSF_). Water concentration in the VOI was estimated using the previously reported values of tissue-specific water concentration ^36^ :

$$\left[ \text{H}\text{2}\text{O} \right]=55.6M\times(0.82 \times f_{GM}+0.73 \times f_{WM}+ f_{CSF})$$

Absolute metabolite concentration in the tissue (i.e. excluding the CSF fraction) was then achieved by using the calculated VOI’s water concentration and partial volume correction:

$\left[ \text{Metabolite} \right]=\text{f}\text{Metab} \times\frac{[\text{H}\text{2}\text{O}]}{(1-\text{f}\text{CSF})}$.

### **Appendix S2: Description of statistical estimators used**

When fitting a set of data points {*y_i_*}^i=1,…,N^ , the nonlinear least-squares fitting algorithm (lsqcurvefit, Matlab 2022b) adjusts a model function to minimize the sum of squares of the difference between the fitted points and the observed points, that is $\sum_{i=1}^{N} \left( y_{i}- \hat{y_{i}} \right)^{2}$.

What we called residuals in this article is actually the absolute value of residuals:

$${Res}_{i}=\left| y_{i}- \hat{y_{i}} \right|$$

The R^2^ coefficient was calculated as:

$$R^{2}=1-\frac{\sum_{i=1}^{N} \left( y_{i}- \hat{y_{i}} \right)^{2}}{\sum_{i=1}^{N} \left( y_{i}- mean(y_{i}) \right)^{2}}$$

To estimate the relevance of a fitting model, we computed the Akaike information criterion (AIC), its corrected counterpart for a large number of degrees of freedom (cAIC) and the Bayesian information criterion (BIC). If *N* is the number of data points (i.e. *N*=51 here), *k* is the number of free variables in the fitting model, and $\sigma^{2}=\frac{1}{N}\sum_{i=1}^{N} \left( y_{i}- \hat{y_{i}} \right)^{2}$ then:

$AIC=N\log\left( \sigma^{2} \right)+2k+N(1+log(2\pi$))

$$cAIC=AIC+\frac{2k(k+1)}{N-k-1}$$

$BIC=N\log\left( \sigma^{2} \right)+k\log\left( N \right)+N(1+log(2\pi$))

The 95% confidence interval on the fitted value of a variable *j* was calculated using the Jacobian *J_f_* calculated by the lsqcurvefit algorithm, as follows:

$${CI}^{95\%}(j)=\pm1.96\sqrt{\frac{1}{N-k}\left( J_{f}^{T}J_{f} \right)_{jj}^{-1}\sum_{i=1}^{N} \left( y_{i}- \hat{y_{i}} \right)^{2}}$$

| **Pool** | **Parameter** | **Initial value** | **Lower bound** | **Upper bound** |
| --- | --- | --- | --- | --- |
| Water | T_1_ | 1.95 s | 1.6 s | 3.5 s |
|  | T_2_ | 32 ms | 20 ms | 45 ms |
|  | ΔB_0_ | 0 ppm | -1.5 ppm | +1.5 ppm |
| MT | δ^MT^ | -2.34 ppm | -2.5 ppm | 0 ppm |
|  | k_ex_^MT^ | 20 Hz | 10 Hz | 60 Hz |
|  | f_H_^MT^ | 5% | 0% | 50% |
| Glutamate | f_H_^Glu^ | 0.02% | 0% | 0.5% |
|  | k_ex_^Glu^ | 1301 Hz (GM) / 1276 Hz (WM) | *Fixed* | *Fixed* |
| Guanidium | f_H_^Guan^ | 0.3% | 0% | 0.7% |
|  | k_ex_^Guan^ | 300 Hz | *Fixed* | *Fixed* |
| Amide | f_H_^APT^ | 3.6% | 0% | 9% |
|  | k_ex_^APT^ | 25 Hz | *Fixed* | *Fixed* |
| Hydroxyl | f_H_^OH^ | 0.4% | 0% | 1.1% |
|  | k_ex_^OH^ | 2000 Hz | *Fixed* | *Fixed* |
| NOE^1^ | f_H_^NOE1^ | 0.4% | 0% | 8% |
|  | k_ex_^NOE1^ | 16 Hz | *Fixed* | *Fixed* |

**Table S1: Fitting model used for quantitative glutamate mapping at B_1_ = 5 µT**

We provide here our own measurements of glutamate’s exchange rate in vitro. Glutamate phantoms were prepared in different conditions. To study pH influence, a set of 40 mM glutamate solutions in PBS at different pH values ranging from 5 to 8 was prepared. Additionally, a set of three 100 mM glutamate solutions in different buffers (MilliQ water, PBS 10 mM, HEPES 10 mM) were prepared at pH = 7.0. For each solution, the pH was adjusted by adding a few drops of NaOH (10%) or HCl (3%). Phantoms were imaged in air-tight tubes at 11.7T with a CEST saturation module of t_sat_ = 1 s, with B_1_ = [3,4,5,6,7,8] µT. Exchange rates were fitted with Bloch-McConnel simulations (including a plausible range of variation on T_1_ and T_2_ values for water) and are indicated below in Tables S2 and S3.


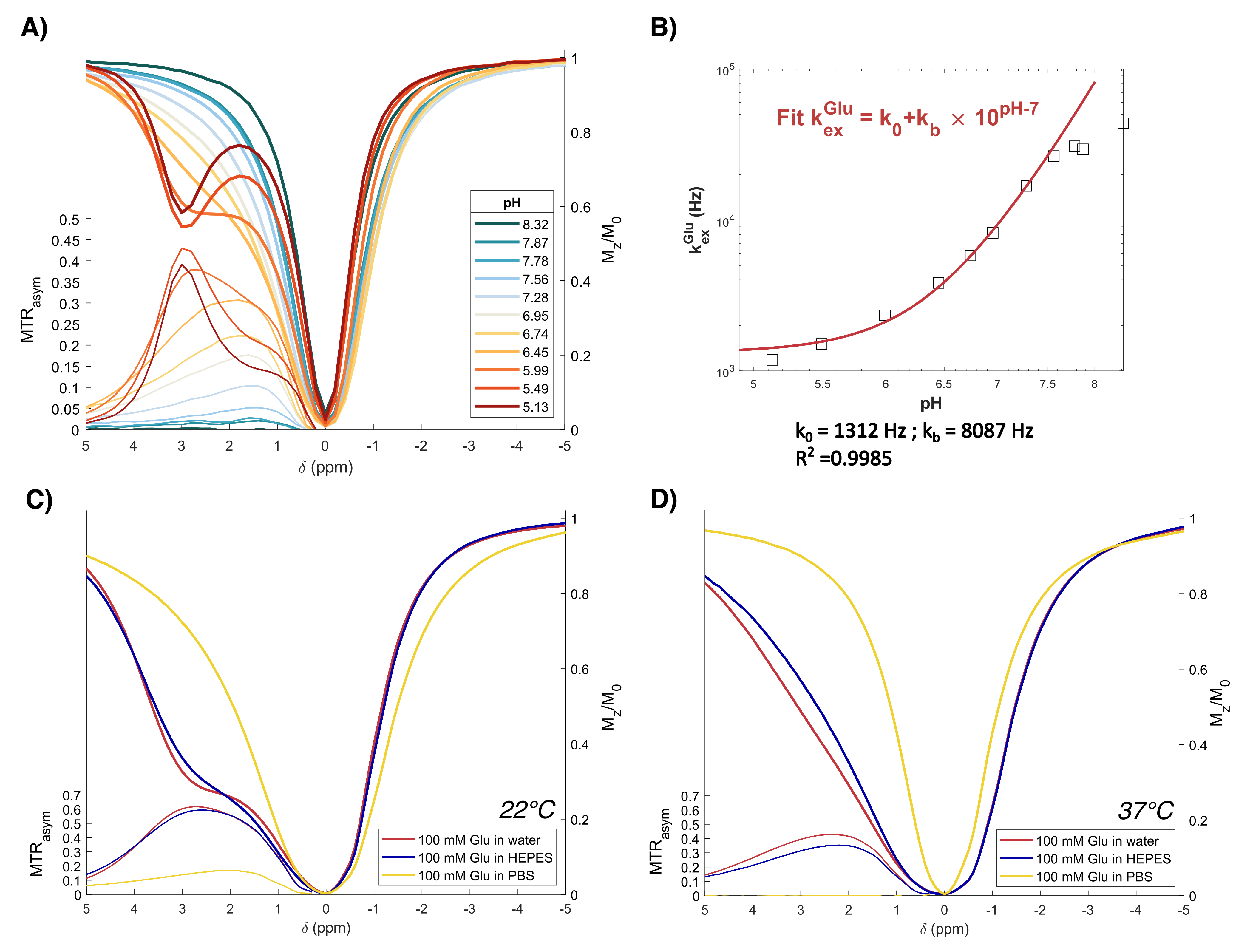


### **Figure S1: Glutamate phantoms: characterization of glutamate exchange rate in vitro**

A) Average Z-spectra measured at B_1_ = 5 µT, t_sat_ = 1 s, in pH-dependent phantoms of 40 mM [Glu]. Measurements were done in a 72 mm quadrature volume coil with a RARE-CEST sequence, using an additional WASSR acquisition (B_1_ = 0.2 µT, δ = -1:0.1:1 ppm) for B_0_ correction.

B) Fitted exchange rate values for each phantom of varying pH, by fitting simultaneously all B_1_ values. The pH dependency was fitted using the base-catalyzed law:

$$k_{ex}^{Glu}=k_{0}+k_{b}{10}^{\text{pH}-7}$$

Values of k_ex_^Glu^ for pH>7.5 were removed from the fit because of inaccuracy in k_ex_^Glu^ evaluation.

C-D) Average Z-spectra measured at B_1_ = 5 µT, t_sat_ = 1 s, of 100 mM [Glu] phantoms at pH = 7.0 in different buffers: milliQ water, 10 mM HEPES and 10 mM PBS at room temperature (C) and at 37°C (D). Measurements were done in a cryoprobe with a LASER-CEST sequence. Phantoms were first scanned at room temperature, then, a circulating water heating system was used to scan them at approximately 37°C (temperature of phantoms were controlled before and at the end of the scan). Fitted exchange rate values are indicated below.

| **Experimentally measured pH** | **Estimated k_ex_^Glu^ (Hz)** | **R^2^** | **CI 95% (Hz)** |
| --- | --- | --- | --- |
| 5.13 | 1182 | 0.9962 | 11 |
| 5.49 | 1508 | 0.9955 | 17 |
| 5.99 | 2331 | 0.9947 | 30 |
| 6.45 | 3828 | 0.9965 | 33 |
| 6.74 | 5809 | 0.9979 | 40 |
| 6.95 | 8207 | 0.9979 | 150 |
| 7.28 | 16808 | 0.9976 | 527 |
| 7.56 | 26480 | 0.9989 | 357 |
| 7.78 | 30779 | 0.9994 | 259 |
| 7.87 | 29393 | 0.9995 | 186 |
| 8.32 | 43795 | 0.9989 | 794 |

### **Table S2: Glutamate exchange rate as a function of pH at room temperature.** Estimations of k_ex_^Glu^ at pH > 7.5 are likely to be inaccurate because of low signal due to fast-exchanging regime.

| **Buffer** | **At 22°C** | | | **At 37°C** | | |
| --- | --- | --- | --- | --- | --- | --- |
|  | **Estimated k_ex_^Glu^ (Hz)** | **R^2^** | **CI 95% (Hz)** | **Estimated k_ex_^Glu^ (Hz)** | **R^2^** | **CI 95% (Hz)** |
| H_2_O MilliQ | 3202 | 0.993 | 19 | 5763 | 0.995 | 25 |
| HEPES 10mM | 3727 | 0.993 | 24 | 7447 | 0.998 | 13 |
| PBS 10mM | 13157 | 0.999 | 3 | *149300* | *0.999* | *4139* |

**Table S3: Glutamate exchange rate as a function of buffer at room temperature and at 37°C.** Estimation of k_ex_^Glu^ in PBS at 37°C is likely to be inaccurate because of low signal due to fast-exchanging regime.

In order to validate our quantitative spectroscopy pipeline, an in vitro study was carried out on two phantom solutions. An initial phantom solution of choline ([Cho] = 2.5 mM), creatine ([Cr] = 15 mM) and glutamate ([Glu] = 15 mM) was made in physiological serum (phantom 1). Half of the solution was collected and then diluted by 2 to make up a second phantom (phantom 2). pH of each solutions was adjusted to 7.2 using a few drops of NaOH (10%) and/or HCl (3%). Phantoms were simultaneously imaged in air-tight tubes at approximately the same distance from the surface of the cryoprobe. Metabolite and water ^1^H-MRS spectra (32 and 16 averages respectively) were acquired in order to be quantified at TE = 20 ms, TR = 6 s in a voxel of 45 µL. To estimate T_2_ values, additional metabolite and water spectra were acquired at TE = [ 20, 35, 50, 95, 145, 200, 300, 400, 500, 600, 700, 800, 900, 1000 ] ms and additionally TE = [1500, 2000, 2500] ms for water. Results are provided in Figure S2. At TE = 20 ms, LCModel fits provided metabolite concentrations with a Cramer-Rao lower bound CRLB<2%. T_2_ values were fitted using numerous TE measurements, more than in the in vivo experiment since no MM spectra are required in vitro. This systematically gave us a fit with R^2^>0.98. Note that for phantoms, T_2_ corrections were of little importance for quantification, considering how they were much higher than TE = 20 ms. Finally on these phantoms, quantitative ^1^H-MRS provided reliable quantification with a maximum of 22% error, and in particular an average error on [Glu] estimation of 8%.

### **
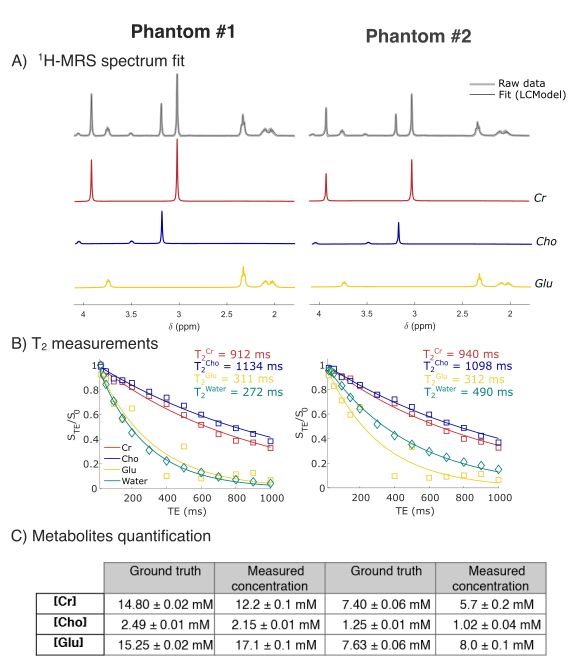
**

### **Figure S2: Validation of quantitative spectroscopy pipeline *in vitro***

A) ^1^H-MRS spectra acquired in both phantoms and LCModel fitting. LCModel basis in this instance included simulated spectra of Cr, Cho and Glu at TE = 20 ms.

B) T_2_ estimation for Cr, Cho, Glu as well as water. A mono-exponential fit was applied to metabolite concentrations outputted by LCModel, and to the measured area under the peak for water spectra. [Glu] measurement at 300 ms was excluded from the fit because LCModel was not able to fit it properly (CRLB > 10%).

C) Quantitative estimations of metabolites concentrations in each phantoms versus ground truth. Ground truth values have been calculated according to the exact mass of compounds measured during the preparation of phantoms, and the indicated error was calculated according to experimental uncertainties over mass and volume. For quantitative estimations, standard deviation over 6 successive measurements of quantitative concentrations were indicated.

| **Pools included in the candidate model** | **R^2^** | **AIC** | **cAIC** | **BIC** |
| --- | --- | --- | --- | --- |
| Water, MT | 0.96211 | -250 | -248 | -240 |
| Water, MT, Glu | 0.97140 | -262 | -260 | -250 |
| Water, MT, Glu, Guan | 0.99225 | -327 | -324 | -313 |
| Water, MT, Glu, OH | 0.98234 | -285 | -282 | -271 |
| Water, MT, Glu, APT | 0.98384 | -289 | -286 | -276 |
| Water, MT, Glu, Tau | 0.97243 | -262 | -259 | -248 |
| Water, MT, Glu, NOE^1^ | 0.97140 | -260 | -257 | -246 |
| Water, MT, Glu, Guan, OH | 0.99402 | -338 | -334 | -322 |
| Water, MT, Glu, Guan, APT | 0.99709 | -375 | -371 | -359 |
| Water, MT, Glu, Guan, Tau | 0.99234 | -325 | -322 | -310 |
| Water, MT, Glu, Guan, NOE^1^ | 0.99226 | -325 | -321 | -309 |
| Water, MT, Glu, Guan, OH, Tau | 0.99402 | -336 | -331 | -318 |
| Water, MT, Glu, Guan, APT, OH | 0.99912 | -433 | -429 | -416 |
| Water, MT, Glu, Guan, APT, NOE^1^ | 0.99759 | -382 | -378 | -365 |
| Water, MT, Glu, Guan, OH, NOE^1^ | 0.99399 | -336 | -331 | -318 |
| Water, MT, Glu, Guan, OH, NOE^1^, NOE^2^ | 0.99401 | -431 | -426 | -412 |
| Water, MT, Glu, Guan, APT, NOE^1^, NOE^2^ | 0.99759 | -380 | -375 | -361 |
| Water, MT, Glu, Guan, APT, OH, Tau | 0.99911 | -431 | -426 | -412 |
| Water, MT, Glu, Guan, APT, OH, NOE^1^ | 0.99958 | -469 | -464 | -450 |
| Water, MT, Glu, Guan, APT, OH, NOE^2^ | 0.99912 | -431 | -426 | -412 |
| Water, MT, Glu, Guan, APT, OH, NOE^1^, Tau | 0.99957 | -467 | -460 | -445 |
| Water, MT, Glu, Guan, APT, OH, NOE^1^, NOE^2^ | 0.99958 | -467 | -460 | -446 |
| Water, MT, Glu, Guan, APT, OH, NOE^1^, Tau, NOE^2^ | 0.99957 | -464 | -456 | -441 |
| Water, MT, Glu, Guan, APT, OH, Tau (k_ex_^Tau^=10000Hz) | 0.99913 | -432 | -427 | -413 |
| Water, MT, Glu, Guan, APT, OH, NOE^1^, Tau (k_ex_^Tau^=10000Hz) | 0.99958 | -467 | -461 | -446 |

**Table S4: All the combinations of pools tested as candidate models**

**
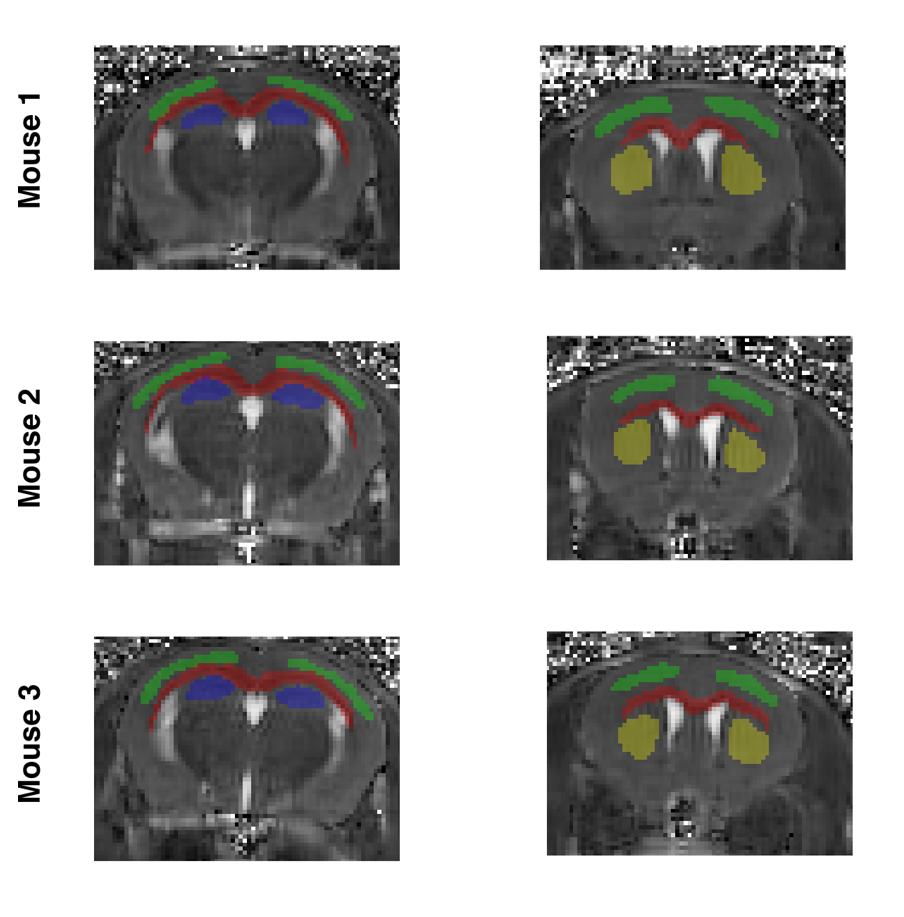
**

**Figure S3: Manually segmented ROIs**

Manually segmented ROIs used to calculate average [Glu] values in several brain regions. Red is corpus callosum, green is cortex, blue is hippocampus and yellow is striatum.

| **Pool** | **Parameter** | **Lower bound** | **Upper bound** |
| --- | --- | --- | --- |
| Water | T1 | 1.5 s | 3.0 s |
|  | T2 | 25 ms | 45 ms |
|  | ΔB_0_ | -1.0 ppm | +1.0 ppm |
| MT | δ^MT^ | Fixed to 0 ppm | Fixed to 0 ppm |
|  | k_ex_^MT^ | 12 Hz | 30 Hz |
|  | f_H_^MT^ | 8% | 13% |
| Glutamate | f_H_^Glu^ | 0.017% | 0.049% |
|  | k_ex_^Glu^ | *Fixed* | *Fixed* |
| Guanidium | f_H_^Guan^ | 0.2% | 0.9% |
|  | k_ex_^Guan^ | *Fixed* | *Fixed* |
| Amide | f_H_^APT^ | 3% | 5.5% |
|  | k_ex_^APT^ | *Fixed* | *Fixed* |
| Hydroxyl | f_H_^OH^ | 0.7% | 1% |
|  | k_ex_^OH^ | *Fixed* | *Fixed* |
| NOE^1^ | f_H_^NOE1^ | 3% | 4.5% |
|  | k_ex_^NOE1^ | *Fixed* | *Fixed* |

### **Table S5: Ranges of variation of parameters used in simulations**


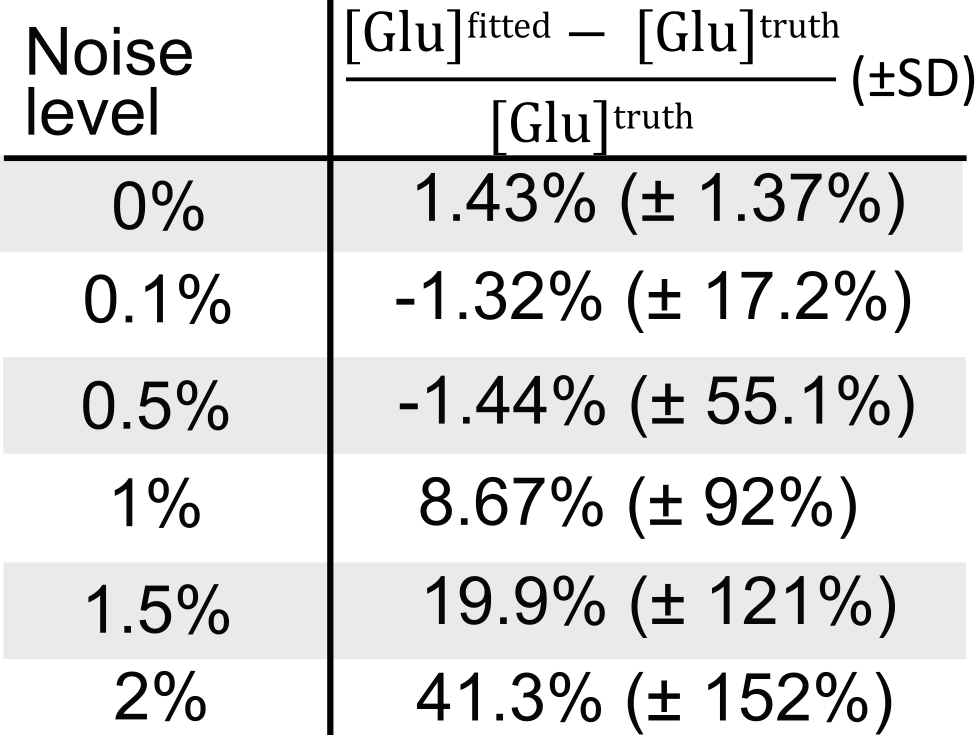


**Table S6: Impact of noise level on the robustness of the fitting model**

Estimations are assessed on a set of 2000 simulations. Outliers, defined as larger than three times the median absolute deviation, were removed from the average.

##
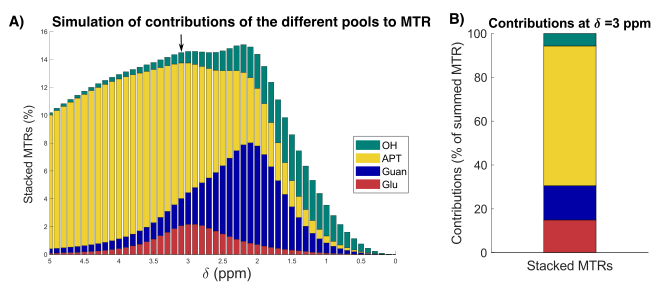


### **Figure S4: Simulation of contributions to MTR for each metabolite in the multi-pool model of Table 3**

A) For each CEST agent, a 3-pool model (water, MT and pool of interest) was computed to evaluate the contribution of each pool on the total MTR at 11.7T, t_sat_ = 1 s, B_1_ = 5 µT. Parameters of the simulation were fixed to what is indicated in Table 2 and the mean striatum results found in table 5, with [Glu] = 6.5 mM. Specific pool contribution to MTR was evaluated by comparing the 3-pool signal to a 2-pool simulation with only water and MT.

B) Estimation of contributions of the different metabolites to MTR explicitly normalized to 3 ppm total sum of MTR. Contribution of metabolite *i* was computed as:

$\text{Contribution}^{i}(\%)=100\times\frac{\text{MTR}^{i}(3ppm)}{\sum_{i} \text{MTR}^{i}(3ppm)}$.
